# Non-malignant conjunctival epithelial masses with ocular surface squamous neoplasia-like optical coherence tomography features

**DOI:** 10.1007/s10792-021-01743-y

**Published:** 2021-03-10

**Authors:** Ágnes Füst, Jeannette Tóth, László Imre, Zoltán Zsolt Nagy

**Affiliations:** 1 Department of Ophthalmology, Semmelweis University, Budapest, Hungary; 2 2nd Department of Pathology, Semmelweis University, Budapest, Hungary; 3 Department of Ophthalmology, Bajcsy Zsilinszky Hospital and Clinic, Budapest, Hungary

**Keywords:** Ocular surface squamous neoplasia, Optical coherence tomography, Conjunctival papilloma, Conjunctival parakeratosis

## Abstract

**Purpose:**

To observe and describe the anterior segment optical coherence tomography features of limbally localised non-malignant epithelial mass lesions

**Methods:**

Thirteen patients (age: 66.9 ± 16.3 years) with conjunctival mass suggesting ocular surface squamous neoplasia with biomicroscopic examination were imaged using anterior segment ocular coherence tomography (anterior segment optical coherence tomography)/Cirrus HD-OCT, Model 4000, Carl Zeiss Meditec, Inc., Dublin, CA, and Spectralis HRA + OCT system, Heidelberg Engineering, Vista, CA/. Cases with ocular surface squamous neoplasia-like anterior segment optical coherence tomography (hyperreflective, thickened epithelium and an abrupt transition from normal to abnormal) were included in the study. Maximal thickness of the epithelium was measured. Histological diagnosis was gained from an excisional or incisional biopsy or impression cytology specimens.

**Results:**

In six patients (age: 68.5 ± 15.4 years) with ocular surface squamous neoplasia-like anterior segment optical coherence tomography features, the histological diagnosis was other than ocular surface squamous neoplasia (papilloma, parakeratosis and a keratotic plaque with mild dysplasia), and ocular surface squamous neoplasia in seven cases (age: 65.6 ± 18.0 years). The maximal epithelial thickness was between 250 and 859 µm in non-ocular surface squamous neoplasia cases and between 252 and 596 µm in ocular surface squamous neoplasia cases.

**Conclusion:**

Non-malignant epithelial lesions can mimic ocular surface squamous neoplasia on anterior segment optical coherence tomography.

## Introduction

Optical coherence tomography (OCT) is a device that uses low-coherence interferometry for generating detailed cross-sectional images in a noncontact way. Although it had been first developed for examining the structure of the retina, anterior segment optical coherence tomography (AS-OCT) soon became available. The axial resolution of the commercially available high-resolution Fourier-domain OCT devices is 5–7μm; however, the mostly custom-built, ultrahigh-resolution OCT is capable of an axial resolution as high as 1–4μm. Both the high-resolution and the ultrahigh-resolution OCT devices are able to produce good-quality image of the layers of the cornea and conjunctiva. Beyond that, not only the thickness of the layers is determined, but the different reflectivity of the diverse pathologies reflects their histological characteristics as well. AS-OCT is used to image lesions like corneal dystrophies, inflammation, deposits in cornea, conjunctival degenerations including pterygium, pinguecula and ocular surface neoplasia. [1]

Ocular surface squamous neoplasia (OSSN) is a comprehensive term for malignant or premalignant masses originating from the epithelium of the conjunctiva or the cornea. According to epidemiological studies, risk factors are older age, male gender, immunocompromised state (HIV infection, AIDS), high UV radiation (closeness to the equator). OSSN usually develops in the interpalpebral area of the conjunctiva, most frequently in the limbus. Corneal OSSN can originate either locally or can spread from the limbal conjunctiva. Its presentation can be leukoplakic, gelatinous, papilliform, nodular, diffuse or a mixture of these. As for clinical behaviour, OSSN is characterised by slow local spread and the development of metastasis is unusual. However, it is prone to local recurrence. The histological spectrum extends from moderate epithelial dysplasia through in situ carcinoma to invasive carcinoma.

The use of ultrahigh-resolution [2] and high-resolution [3] AS-OCT in the diagnosis and differential diagnosis of OSSN goes back to 2011 and 2015, respectively. The reported OCT features of OSSN are thickened hyperreflective epithelium with an abrupt transition from the normal epithelium. The cut-off value of maximal epithelial thickening which differentiates between OSSN and normal epithelium was found to be 120 µm [3] in one study and 142µm [4] in another. AS-OCT was shown to be able to discriminate OSSN from other ocular surface pathologies, like various corneal dystrophies and degenerations [5], conjunctival degenerations like pterygium, pinguecula and masses like lymphoma and melanocytic lesions like naevi [3]. The common feature of these lesions is that none of them is intraepithelial, like OSSN. An exception is conjunctival intraepithelial melanocytic hyperplasia, but it is not a mass lesion, and it is not accompanied by the thickening of the epithelium. AS-OCT can detect OSSN on top of other pathologies like ocular rosacea, limbal stem cell deficiency, ocular pemphigoid or scarring [6].

The purpose of this study was to observe and compare the AS-OCT features of limbally located non-malignant and malignant epithelial mass lesions.

## Methods

### Patients

This retrospective study was conducted at our Corneal and Ocular Surface Diseases tertiary referral centre. We searched through the files of patients from 2017 to 2019 who were referred with limbal ocular surface mass suspect for OSSN. Inclusion criteria were set on the base of biomicroscopic and AS-OCT examination and the result of the pathological examination. Patients were included in the study if OSSN features (thickened hyperreflective epithelium with an abrupt transition from normal epithelium, epithelial thickness >142µm [4]) were present in AS-OCT images, but the pathological evaluation excluded OSSN. As control, those pathologically proven OSSN patients were selected who were diagnosed during the same period, examined with the same AS-OCT devices and had the characteristic AS-OCT features.

### Diagnostic procedures

#### Anterior segment optical coherence tomography

AS-OCT imaging was performed with one of the two different high-resolution Fourier-domain OCT devices depending on the availability at the time of the examination: Cirrus HD-OCT, Model 4000, Carl Zeiss Meditec, Inc., Dublin, CA, and Spectralis HRA + OCT system, Heidelberg Engineering, Vista, CA. The records from the affected conjunctival, limbal and corneal areas were taken with AS 5 line Raster and/or AS Cube 512x128 protocols. Care was taken to cover the whole lesion, with supplementary scans taken from different parts, if necessary. The epithelial thickness and reflectivity and the lateral and basal edge of the diseased epithelium were evaluated along with the subepithelial structures. The maximal epithelial thickness was determined with the help of a distance-measuring tool built into the software. The evaluation and the measurements were performed in all of the cases by the same person.

#### Histologic analysis

Excisional or incisional biopsy was performed in all but one case (case No.5), see “Results”, where no removal was decided. In this case, impression cytology was taken. Biopsy specimens were fixed in 10% buffered formalin, dehydrated and embedded in paraffin blocks. The blocks were sectioned at 5µm. Slides and the impression cytology sample were stained with hematoxylin–eosin. In cases where malignancy was not unequivocal, immunohistochemical labelling for the Ki-67-es proliferation marker was performed to detect the rate of proliferating cells.

## Results

Six patients with OSSN-like AS-OCT features had benign lesions. The control group consisted of seven OSSN patients. The mean age of patients with benign pathology and with OSSN was 68.5±15.4 and 65.6±18.0 years, respectively. For detailed demographic data, see Table 1.

**Table 1:** Demographic data of the patients, diagnosis and maximal epithelial thickness

Case No.	Age (year)	F/M	Source of pathology sample	Clinical diagnosis	Pathological diagnosis	Maximal epithelial thickness (μm)
B1	75	F	EB	Conjunctival papilloma or OSSN	Conjunctival papilloma	859
B2	87	M	EB	OSSN	Solar elastosis with parakeratosis	407
B3	62	M	EB	Leukoplakic OSSN	Keratotic plaque	642
B4	73	M	EB	Pinguecula with leukoplakia or leukoplakic OSSN	Pinguecula with epithelial dysplasia	340
B5	73	M	IC	Recurrent herpetic keratitis with epithelial thickening suspect to OSSN	Regular epithelium	271
B6	41	M	EB	Pinguecula or gelatinous OSSN	Stromal degeneration with parakeratosis	250
M1	74	F	IB	Diffuse OSSN	OSSN–CIN	252
M2	80	M	EB with sclerokeratoplasty	Invasive squamous cell carcinoma	Invasive squamous cell carcinoma	370
M3	55	M	EB	OSSN recurrence	OSSN–CIN	548
M4	75	F	EB	OSSN	OSSN–in situ carcinoma	491
M5	70	M	EB	OSSN recurrence	OSSN–CIN	345
M6	76	M	EB	OSSN on the top of pterygium	OSSN–CIN	596
M7	29	M	EB	OSSN	OSSN–CIN	435

F: female, M: male, EB: excisional biopsy, IB: incisional biopsy, IC: impression cytology, OSSN: ocular surface squamous neoplasia, CIN: conjunctival intraepithelial neoplasia

**Fig. 1:**
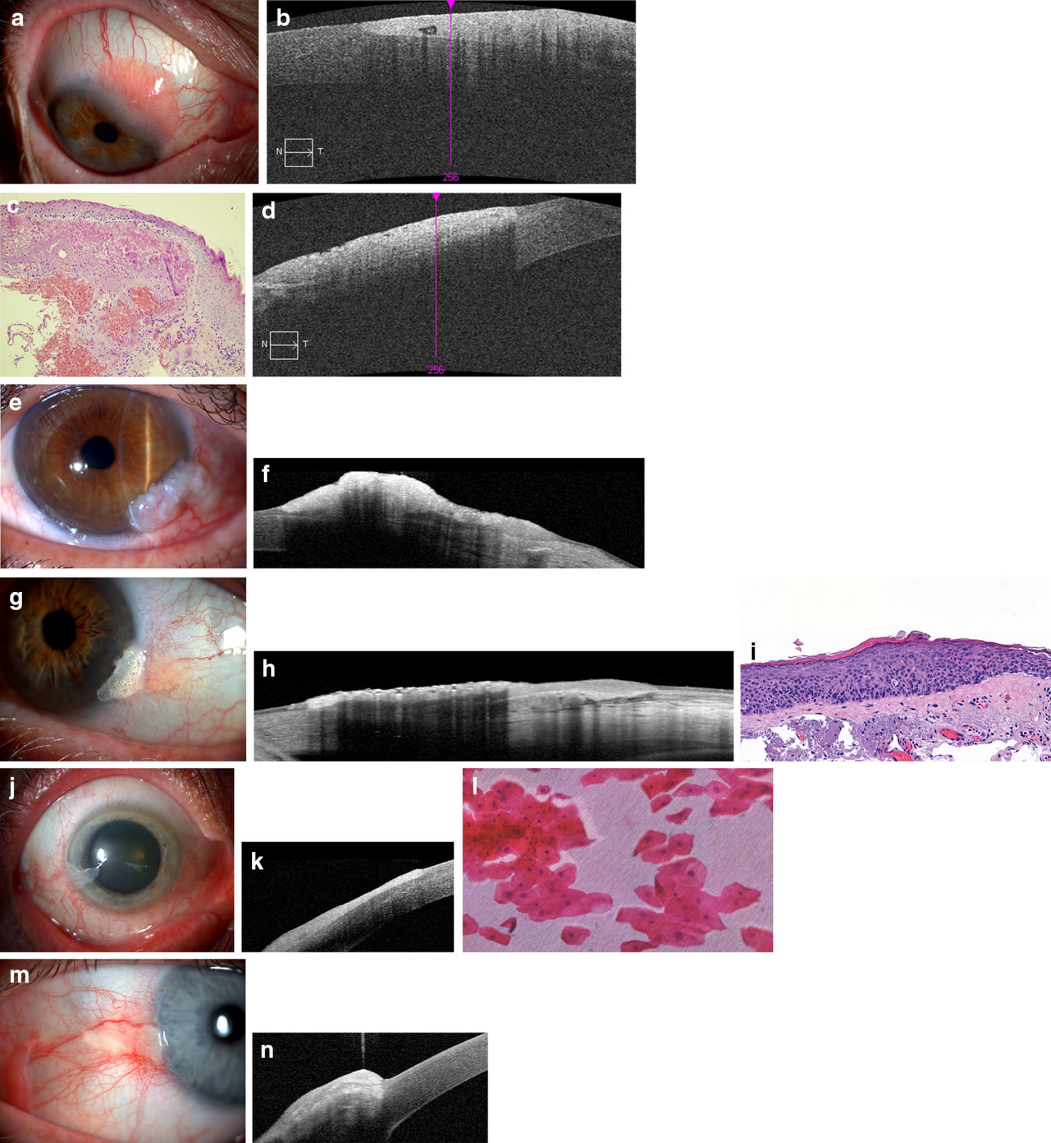
Clinical photographs, AS-OCT images and histology sections of the benign cases. **a**, **b**: Case B1: conjunctival papilloma. **a:** slit lamp photograph, **b**: AS-OCT image. **c**, **d**: Case B2: parakeratosis and solar elastosis. **c**: histology, HE, x20. **d**: AS-OCT image. **e**, **f**: Case B3: keratotic plaque with signs of moderate epithelial dysplasia. **e**: slit lamp photograph, **f**: AS-OCT image. **g**, **h**, **i:** Case B4: pinguecula with mild epithelial dysplasia on the surface. **g**: slit lamp photograph, **h**: AS-OCT image, **i**: histology, HE, obj. x20. **j**, **k**, **l**: Case B5: recurrent herpetic keratitis with epithelial thickening **j**: slit lamp photograph, **k**: AS-OCT image, **l**: impression cytology, HE, obj. x20. **m**, **n**: Case B6: parakeratosis over stromal degeneration. **m**: slit lamp photograph, **n**: AS-OCT image

The source of pathology specimen and the diagnosis of the 13 cases are included in Table 1. The six benign cases are described in detail below; their AS-OCT images are presented in Fig. 1.

### Case reports

Case B1: The patient suffered from long-term inflammation of the right eye refractory to topical antibiotic therapy. By slit-lamp examination, a papilliform limbal mass was found in the superior nasal quadrant (Fig. 1a). On AS-OCT (Fig. 1b) inside the thickened hyperreflective epithelium, some vessels were observed. The excisional biopsy specimen was histologically a conjunctival papilloma.

Case B2: The patient was referred for a limbal conjunctival mass on the right eye. He had phacoemulsification 3 years before and vitrectomy 17 years before in this eye and multiple intravitreal bevacizumab injections for macular degeneration in the contralateral eye. By biomicroscopy, a 2 clock hour limbal papilliform, partly vascularised mass was detected in the temporal inferior quadrant. The eye was quiet. Pathological examination (Fig. 1c) of the excisional biopsy showed parakeratotic, slightly hyperplastic epithelium without signs of atypia. There was an extensive solar elastosis of collagen fibres in the stroma. The diagnosis was solar elastosis with parakeratosis.

Case B3: The patient presented with a limbal lesion of her left eye. The lesion that has been growing for two months and which occupied the 3 to 5 o’clock position was 3 mm wide. The nodular mass was localised mostly on the surface of the cornea (Fig. 1e). Microscopy of the excisional biopsy disclosed a hyper- and parakeratotic, markedly hyperplastic epithelium, with an abrupt transition to the normal epithelium. The epithelium was acanthotic, with slight spongiosis. Dyskeratotic cells, numerous apoptotic cells and several mitoses could be observed. The epithelial basement membrane was intact, and severe solar elastosis was found in the stroma. The diagnosis was keratotic plaque with signs of moderate epithelial dysplasia.

Case B4: The patient was referred because of a white limbal deposit on the left eye which could be wiped off but regrew every time. We saw a leukoplakic lesion over the limbal area with sharp edges beside a yellowish thickening of the conjunctiva (Fig. 1g). Pathological examination (Fig. 1i) revealed hyperkeratotic hyperplastic epithelium with mild dysplasia in some foci. Severe solar elastosis was found in the subconjunctival collagen fibres. The diagnosis was pinguecula with mild epithelial dysplasia on the surface.

Case B5: The patient was treated for chronic keratouveitis with secondary open-angle glaucoma of the right eye for 2 months. His treatment was ganciclovir gel and fluorometholone eye drops. At slit-lamp, mild cilio-conjunctival injection was found with a flat vascularised tissue overgrowth on the surface of the cornea between the 8 and 9 o’clock positions (Fig. 1j). On the corneal surface, this spread centrally in a subepithelial linear haze. The stroma was otherwise clear, but there were precipitates on the posterior corneal surface. No cells were seen in the anterior chamber. Corneal sensitivity was decreased. As the clinical presentation and the AS-OCT images (Fig. 1k) suggested OSSN, impression cytology (Fig. 1l) was performed. No malignant cells were detected. The treatment was continued and one month later the inflammatory signs faded, and the overgrowth on the cornea became much thinner and vascularisation decreased.

Case B6: The patient presented with chronic pingueculitis in the nasal side of the left eye that lasted for half a year. As the limbal area had a pronounced gelatinous appearance (Fig. 1m), AS-OCT was performed (Fig. 1n). Pathological examination revealed parakeratotic epithelium over degenerated stromal structure consistent with a pingueculum.

**Fig. 2:**
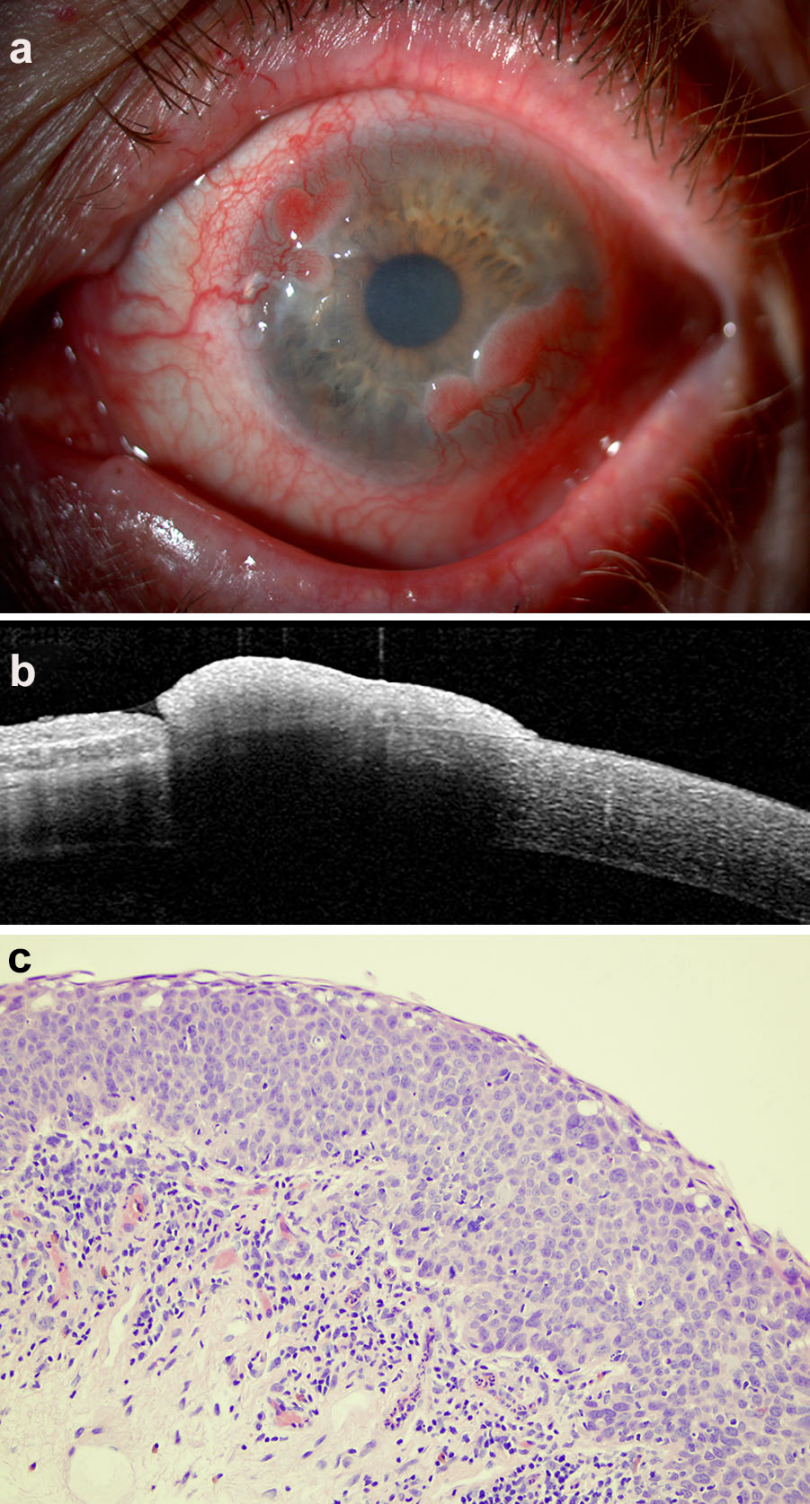
Case M4: OSSN–in situ carcinoma. **a**: slit lamp photograph, **b**: AS-OCT image, **c**: histology, HE, x20

Out of the seven OSSN patients, conjunctival intraepithelial neoplasia (CIN) occurred in five, one further patient had carcinoma in situ (Fig. 2) and one had invasive squamous cell carcinoma with a scleral invasion at the limbus.

### Maximal epithelial thickness

The mean maximal epithelial thickness was 461 ± 241 μm and 434 ± 121 μm in the benign group and OSSN group, respectively. The range spread from 250 μm to 859 μm in the benign group and from 252 μm to 596 μm in the OSSN group

## Discussion

It is uniformly accepted that the thickened hyperreflective epithelium with an abrupt transition to normal epithelium is the sign of OSSN in high-resolution or ultrahigh-resolution AS-OCT images [3, 7, 8]. The specificity of AS-OCT imaging was found 100% for differentiating OSSN from other ocular surface pathologies [3–6]. In a recent article [9], the AS-OCT features of conjunctival papilloma were described as thickened hyperreflective epithelium with or without an abrupt transition to the normal epithelium, and dome-shaped or lobulated configuration, besides a highly vascularised core. However, except for the latter article, to our best knowledge, our study is the first which addresses the AS-OCT examination of non-malignant lesions causing epithelial hypertrophy. In our study, six such cases were presented. The diagnoses were conjunctival papilloma, stromal degeneration with parakeratosis, keratotic plaque, pinguecula with epithelial dysplasia and recurrent herpetic keratitis.

It is not surprising that not only OSSN but the benign lesions we examined are presenting themselves as epithelial thickenings, too. Neither the abrupt edge nor the measure of hypertrophy (≥250 um) is surprising when looking at the clinical pictures (Fig. 1) and the natures of the diseases. The inner reflectivity of the entities on AS-OCT is determined qualitatively in everyday clinical practice. It can be increased, decreased or can be identical, compared to the normal, depending on their microscopic structural differences [1]. However, both the benign and the malign epithelial hypertrophies have a compact histological structure, independent from their cellular composition. This means that it is very likely that their reflectivity is similarly high.

Currently, the accepted therapy for OSSN is either surgical—with or without adjuvant local chemotherapy—or medical, where primary local chemotherapy is applied alone. In the latter case, the diagnosis is set up on the basis of clinical examination combined with some kind of imaging technics, most frequently the AS-OCT, and no biopsy for histological examination is performed [10]. However, the results of the current paper show that some lesions are indistinguishable from OSSN by the combination of slit-lamp examination and AS-OCT. Local chemotherapy with mitomycin-C, interferon alpha 2b or 5-fluorouracil is not without the danger of severe toxic side effects like limbal stem cell deficiency, and severe ocular surface inflammation or allergic reaction. This raises the question of whether the pathological examination can be omitted when we intend to treat the patient with primary chemotherapy.

One limitation of our study is that two different high-resolution Fourier-domain OCT devices were used: Cirrus HD-OCT (Zeiss) and Spectralis HRA + OCT system (Heidelberg), because the former became defective after examination of the first patients, and the latter became available only after the former had gone wrong. However, patients from both benign and control groups were among the persons who were examined with the former device as well as among those who were examined with the latter one. Moreover, there were only minor quality differences between the two machines, and the examined features were well detectable with both devices. Other limitations are the relatively small number of cases and the retrospective nature. 

In conclusion, non-malignant epithelial lesions, such as papilloma, parakeratosis and keratotic plaque, can mimic ocular surface squamous neoplasia on anterior segment optical coherence tomography. As a consequence, inclusion of some extra OCT features for the characterisation of OSSN or combination of the OCT with other examination methods like impression cytology, in vivo confocal microscopy or OCT angiography may be necessary to render the diagnosis more specific.

## Data Availability

Data are available at the authors.
